# Neuroprotective effects of erythropoietin on rat retinas subjected to oligemia

**DOI:** 10.6061/clinics/2018/e161

**Published:** 2018-04-09

**Authors:** Litia Alves de Carvalho, Renata Fleming, Moysés Sant’Anna, Roberta Guimarães, Adalmir Morterá Dantas, Eduardo Morizot-Leite, Leny A. Cavalcante, Silvana Allodi

**Affiliations:** I Programa de Neurobiologia, Instituto de Biofisica Carlos Chagas Filho, Universidade Federal do Rio de Janeiro, Rio de Janeiro, RJ, BR; II Experimental Therapeutics and Molecular Imaging Laboratory, Neuroscience Center, Department of Neurology, Massachusetts General Hospital, Boston, Massachusetts, USA; III Program in Neuroscience, Harvard Medical School, Boston, Massachusetts, USA; IV Hospital Universitário Clementino Fraga Filho, Faculdade de Medicina, Universidade Federal do Rio de Janeiro, Rio de Janeiro, RJ, BR; V Instituto Benjamin Constant, Rio de Janeiro, RJ, BR

**Keywords:** Hematopoietic Growth Factor, Retinal Ganglion Cells, Glial Cells, Neuroprotection, Bilateral Common Carotid Artery Occlusion

## Abstract

**OBJECTIVES:**

Erythropoietin may have neuroprotective potential after ischemia of the central nervous system. Here, we conducted a study to characterize the protective effects of erythropoietin on retinal ganglion cells and gliotic reactions in an experimentally induced oligemia model.

**METHODS:**

Rats were subjected to global oligemia by bilateral common carotid artery occlusion and then received either vehicle or erythropoietin via intravitreal injection after 48 h; they were euthanized one week after the injection. The densities of retinal ganglion cells and contents of glial fibrillary acidic protein (astrocytes/Müller cells) and cluster of differentiation 68 clone ED1 (microglia/macrophages), assessed by fluorescence intensity, were evaluated in frozen retinal sections by immunofluorescence and epifluorescence microscopy.

**RESULTS:**

Retinal ganglion cells were nearly undetectable one week after oligemia compared with the sham controls; however, these cells were partially preserved in erythropoietin-treated retinas. The contents of glial fibrillary acidic protein and cluster of differentiation 68 clone ED1, markers for reactive gliosis, were significantly higher in retinas after bilateral common carotid artery occlusion than those in both sham and erythropoietin-treated retinas.

**CONCLUSIONS:**

The number of partially preserved retinal ganglion cells in the erythropoietin-treated group suggests that erythropoietin exerts a neuroprotective effect on oligemic/ischemic retinas. This effect could be related to the down-modulation of glial reactivity, usually observed in hypoxic conditions, clinically observed during glaucoma or retinal artery occlusion conditions. Therefore, glial reactivity may enhance neurodegeneration in hypoxic conditions, like normal-tension glaucoma and retinal ischemia, and erythropoietin is thus a candidate to be clinically applied after the detection of decreased retinal blood flow.

## INTRODUCTION

Erythropoietin (EPO) is a glycoprotein secreted in response to hypoxia that stimulates the differentiation and proliferation of erythrocyte progenitor cells and may have additional physiological effects as a cytokine with antiapoptotic, neurotrophic, angiogenic and antioxidant roles 1-4. The neuroprotective potential of EPO has been described in several animal models of central nervous system (CNS) injury. In murine models of focal cerebral ischemia induced by permanent occlusion of the middle cerebral artery, the chronic intraventricular administration of EPO 24 hours before the ischemic injury reduced the size of the brain lesion area compared to that in animals that received no preconditioning 1. In brain damage by freezing, intraperitoneal EPO administration reduced behavioral disorders, cognitive dysfunction, and brain atrophy for more than eight months after injury 5. Systemic EPO administration after the development of spinal cord lesions decreased the rate of apoptosis in dorsal root ganglion cells 6. However, the exact mechanisms underlying these neuroprotective effects are not yet fully elucidated.

Because the retina may also be affected by degenerative events like those in other parts of the CNS and considering the neuroprotective potential of EPO as a treatment for pathological retinal conditions 7-9, interest in investigating the effects of exogenous EPO administration in various animal models of retinal degeneration has grown. Herein, we report for the first time an evaluation of the neuroprotective potential of EPO after the induction of retinal ischemia in rats using the bilateral common carotid artery occlusion (BCCAO) technique, which mimics human retinopathy caused by the occlusion of this artery or its branches 10.

## MATERIALS AND METHODS

### 1. Oligemia model

Protocols for animal use were approved by the Ethics Committee for Use of Experimental Animals at the Federal University of Rio de Janeiro (UFRJ, process IBCCF020). Twenty young-adult male Wistar rats (aged 3 months) were anesthetized with a mixture of ketamine (70 mg/kg) and xylazine (8 mg/kg, intraperitoneal). After making a cervical medial and ventral incision, the common carotid artery was bilaterally exposed and tied at two levels 2 mm apart from each other. The arteries were sectioned between the two ties.

Sham animals (n=5) underwent artery exposure but were not subjected to artery tying and sectioning. During anesthesia, the body temperatures of the rats was maintained at 37-38°C with a heated blanket.

### 2. EPO treatment

After surgery, all rats were housed in individual cages with easy access to food and water and received tramadol (200 µg/kg, subcutaneous) and enrofloxacin (7.5 mg/kg, subcutaneous). Two days later, a group of 10 rats received either an intravitreal injection of either 1 μl of phosphate buffered saline (PBS) or 1 μl of EPO (400 μg diluted in PBS). Five additional rats were only subjected to BCCAO as a control and received no intravitreal injection.

### 3. Histology, immunofluorescence, cell counting and quantification of fluorescence intensity

Ten days after surgery, sham and experimental animals were euthanized with an overdose of pentobarbital. Their eyes were then removed and opened, and the posterior portions were fixed with 4% paraformaldehyde in phosphate buffer (PB). These portions were cryoprotected in PB with 30% sucrose overnight and sectioned (12-µm thickness). The sections were collected on glass slides pre-coated with poly-L-Lysine. Next, a portion of the material obtained was processed for routine histology to observe the general architecture. Images were acquired with a Carl Zeiss Axioimager M2 microscope.

For immunofluorescence 11, the following primary antibodies were used: mouse anti-Brn3a (retinal ganglion cells, RGC) 12, 1:100, Millipore, USA), rabbit anti-GFAP (astrocytes/Müller cells; 1:100, Millipore) and mouse ED1 (microglia/macrophages, 1:100, Millipore). The secondary antibodies used were anti-mouse conjugated to Alexa 488 and anti-rabbit conjugated to Alexa 647 (1:400; Life Technologies, USA).

Every other retinal serial section obtained after cryotomy was used. Cell bodies in the central retina located approximately 1 mm from the optic disc labeled with brain-specific homeobox/POU domain protein 3A (Brn3a), glial fibrillary acidic protein (GFAP) or cluster of differentiation 68 clone ED1 (ED1) were counted using a microscope (Carl Zeiss Axioimager M2) with an EC-Plan Neofluar 40×/1.30 Oil DIC M27 objective.

**Figure 1:**
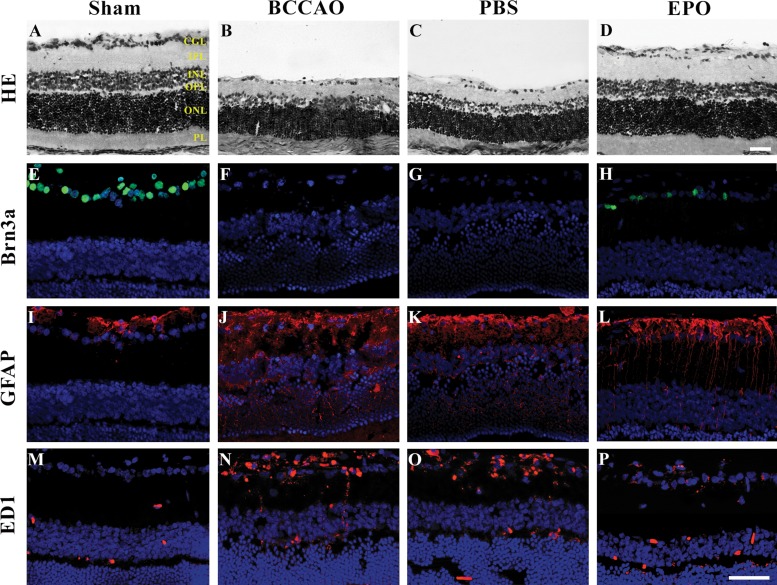
Representative micrographs of retinal sections. (A-P). (A-D) Hematoxylin and eosin (H&E)-stained retinal sections of control retinas (sham), (B) retinas subjected to oligemia by bilateral common carotid artery occlusion (BCCAO), (C) retinas subjected to BCCAO and treated with PBS (PBS), and (D) retinas subjected to BCCAO and treated with erythropoietin (EPO). Note the prominent swelling of neuronal cell bodies and shrinkage of the inner nuclear layer (INL), inner plexiform layer (IPL) and ganglion cell layer (GCL) in B and C. (E-H) Retinal sections immunoreacted with Brn3a showing RGC-positive cells (green) under different conditions: (E) Sham, (F) BCCAO only, (G) BCCAO with PBS treatment, and (H) EPO-treated retinas ten days after oligemia. Brn3a expression is restricted to the GCL and, although labeling is less evident, more Brn3a-labeled cells are present in EPO retinas (H) than in BCCAO (F) or PBS (G) retinas. (I-P) Retinal sections immunoreacted with glial markers (GFAP (I-L) and ED1 (M-P), red) under different conditions: (I,M) Sham, (J,N) BCCAO only, (K,O) PBS, and (L,P) EPO retinas ten days after oligemia. In sham retinas, GFAP-positive cells are more restricted to the GCL, and ED1-positive cells are barely detectable. In both BCCAO (J,N) and PBS retinas (K,O), GFAP- and ED1-positive cells extend throughout the entire retinal thickness, while the labeling is less evident in EPO retinas (L,P) than in the previous conditions, indicating decreased glial reactivity and inflammation ten days after oligemia. Scale bars. 50 µm. Abbreviations: PL, photoreceptor layer; ONL, outer nuclear layer; OPL, outer plexiform layer; INL, inner nuclear layer; IPL, inner plexiform layer; and GCL, ganglion cell layer.

To quantify the fluorescence emission intensities of the GFAP and ED1 markers, the originally acquired image data were used. Mean intensity levels (mean intensity profile, ImageJ imaging software) were calculated from the central retinal areas and expressed as pixel gray-scale values. To compare the overall fluorescence intensities of the retina tissue samples, mean fluorescence intensities were calculated from the selected comparable image areas displaying the central retina as the typical histological structure (sham retina, Fig. 1A). Images for each experimental condition were acquired using identical microscopic image settings. The mean fluorescence emission intensities of the negative controls (with primary antibody suppression) were defined as the negative control reference value, and the mean fluorescence intensities of the sections were set after the treatments.

Statistical analyses were performed using GraphPad Prism software (version 5.0.0.288). Data are expressed as the mean ± standard error of the mean (SEM) and were analyzed by one-way ANOVA followed by the Student-Newman-Keuls test. P values <0.05 were considered significant.

## RESULTS

Ten days after the rats were subjected to oligemia, retinas of both the BCCAO and PBS groups showed reduced thickness compared with that of the sham group, and both plexiform layers appeared to be disorganized (Figure 1 A-C). However, treatment with EPO preserved both the retinal thickness and the organization of the layers. Furthermore, the number of RGCs in the EPO group was significantly increased compared to that in the other groups that underwent BCCAO surgery, although the amount was not similar to that in the sham group (Figure 1D).

**Figure 2:**
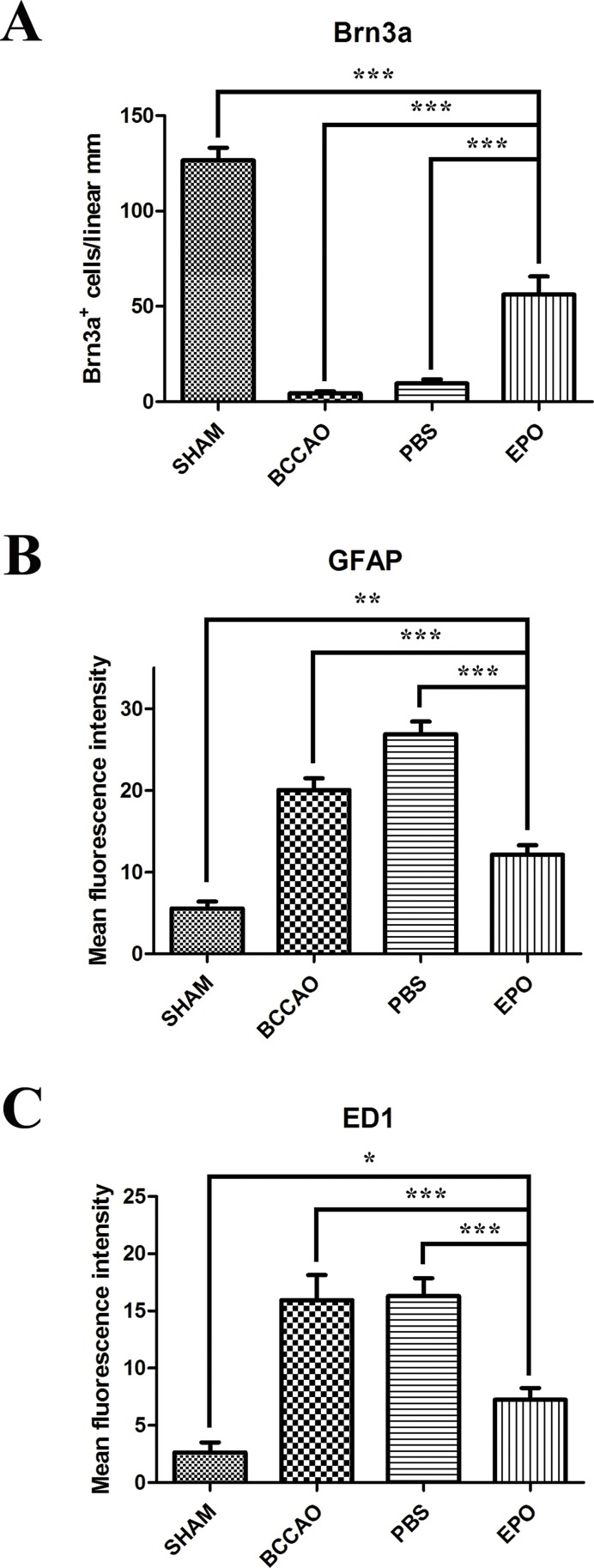
Effects of EPO intravitreal injection on retinal ganglion cell (RGC) density and retinal glial reactivity after oligemia. (A) Quantification of Brn3a-positive cell bodies in retinas subjected to different conditions confirming that the Brn3a protein content decreases to a lesser extent in EPO retinas than in both BCCAO-only and PBS retinas compared with that in sham retinas. Quantification of the fluorescence emission intensity of anti-GFAP (B) and ED1 (C) markers, presented as the mean fluorescence intensity/region of interest (ROI), in retinas subjected to different conditions. Data are expressed as the mean ± standard error of the mean (SEM). *p<0.05; **p<0.005 and ***p<0.001 values were considered significant.

Compared with that in the sham group, Brn3a labeling revealed a significant loss of RGCs in both the BCCAO and the PBS groups (Figure 1E-G) and labeling of a considerable number of cells after EPO administration (Figure 1H). Quantification of Brn3a-positive RGCs showed that 10 days after surgery, BCCAO retinas contained fewer RGCs than those of the sham group. Retinas receiving PBS also showed a substantially reduced number of Brn3a-positive cells compared with that in the sham group. However, retinas treated with EPO (48 h after surgery) had 57% of their RGCs preserved compared to that in BCCAO retinas (Figure 2A).

After retinal trauma, reactive gliosis occurs, as revealed by the overexpression of GFAP 13,14. Therefore, to determine whether EPO could serve as an effective modulator of glial reactivity, we analyzed the fluorescence pattern of GFAP in the retinas of rats subjected to BCCAO. Retinas of BCCAO and PBS group rats showed increased GFAP labeling compared with those of sham group rats (Figure 1I-K). However, retinas treated with EPO showed reduced GFAP labeling (Figure 1L) compared with that in sham retinas. Quantification of fluorescence showed that retinas of the BCCAO and PBS groups (especially the latter) had either more astrocytes/Müller cells or were more reactive to the injury (Figure 2B). Additionally, fluorescence in EPO-treated retinas was decreased to a larger extent compared with that in the BCCAO and PBS groups and was increased compared with that in the sham group (Figure 2B).

Similarly, reactive microglia have been detected in the retina and optic nerve after injury and shown to contribute to neuronal death 15-17. Immunofluorescence labeling of ED1, which is usually upregulated in reactive microglia/macrophages 18, revealed the presence of many more cells in both the BCCAO and PBS groups compared with those in the sham and EPO groups (Figure 1 M-P). Finally, quantification of fluorescence labeling showed that retinas of both the BCCAO and PBS groups had more microglia/macrophages than the sham and EPO groups. Although there was a difference in the number of sham and EPO retinas, it was not as large as the increased fluorescence intensities in both the BCCAO and PBS groups (Figure 2C).

## DISCUSSION

In this study, we showed that EPO injection into the vitreous chamber of oligemia model rats allowed maintenance of about half the number of RGCs and reduced both the macroglial reactivity and the number of retinal microglia/macrophages.

In humans, the inner retina is very sensitive to hypoxia or other insults 19. Because EPO is present in the retina 19,20, several studies have examined its potential neuroprotective role in the visual system 21-29. However, although EPO has been used to treat ocular disorders, concerns as to whether it worsens pathogenicity or provides protection against eye diseases have been raised. For a review, see 30.

In this study, the reduction of RGCs after BCCAO mimicked insults that affect the human eye, such as hypoxia resulting from glaucoma or retinal ischemia 31,32. Although astrocytes and Müller cells are known to produce and secrete neuroprotective factors, and Müller cells are the main source of EPO in the normal retina 20, our data suggest that RGCs are rescued by intravitreal EPO injection. We observed a strong reactivity to GFAP, which may indicate that both Müller cells and astrocytes respond to BCCAO. Additionally, the increased number of reactive microglia/macrophages indicates that a reactive mechanism occurs after BCCAO.

In humans, promising results have been reported using systemic and intravitreal EPO for different ocular conditions (33,34; for a review see 35), and understanding its effects is important for developing better therapies for other pathological retinal conditions. The BCCAO model, used here for the first time to evaluate the effects of EPO on the retina, is useful because it creates an experimental condition comparable to normal tension 36. This model also mimics narrowing of the internal carotid artery, a common and asymptomatic condition of systemic arteriosclerosis 37.

## AUTHOR CONTRIBUTIONS

Carvalho LA designed the study and aided with data collection and management and writing the manuscript. Flemming R, Sant’Anna M, and Guimarães R aided data collection. Dantas AM aided with fundraising and data discussion. Morizot-Leite designed the study. Cavalcante LA discussed the study and aided with fundraising and writing the manuscript. Allodi S aided with fundraising, managing the data, and writing the manuscript.
